# Methods to accelerate fracture healing – a narrative review from a clinical perspective

**DOI:** 10.3389/fimmu.2024.1384783

**Published:** 2024-06-07

**Authors:** Bergita Ganse

**Affiliations:** ^1^ Innovative Implant Development (Fracture Healing), Clinics and Institutes of Surgery, Saarland University, Homburg, Germany; ^2^ Department of Trauma, Hand and Reconstructive Surgery, Clinics and Institutes of Surgery, Saarland University, Homburg, Germany

**Keywords:** trauma, injury, bone, smart implant, growth factors, parathyroid hormone, biomechanics, electromagnetic stimulation

## Abstract

Bone regeneration is a complex pathophysiological process determined by molecular, cellular, and biomechanical factors, including immune cells and growth factors. Fracture healing usually takes several weeks to months, during which patients are frequently immobilized and unable to work. As immobilization is associated with negative health and socioeconomic effects, it would be desirable if fracture healing could be accelerated and the healing time shortened. However, interventions for this purpose are not yet part of current clinical treatment guidelines, and there has never been a comprehensive review specifically on this topic. Therefore, this narrative review provides an overview of the available clinical evidence on methods that accelerate fracture healing, with a focus on clinical applicability in healthy patients without bone disease. The most promising methods identified are the application of axial micromovement, electromagnetic stimulation with electromagnetic fields and direct electric currents, as well as the administration of growth factors and parathyroid hormone. Some interventions have been shown to reduce the healing time by up to 20 to 30%, potentially equivalent to several weeks. As a combination of methods could decrease the healing time even further than one method alone, especially if their mechanisms of action differ, clinical studies in human patients are needed to assess the individual and combined effects on healing progress. Studies are also necessary to determine the ideal settings for the interventions, i.e., optimal frequencies, intensities, and exposure times throughout the separate healing phases. More clinical research is also desirable to create an evidence base for clinical guidelines. To make it easier to conduct these investigations, the development of new methods that allow better quantification of fracture-healing progress and speed in human patients is needed.

## 1 Introduction

Fracture healing is a lengthy process that, in humans, usually takes several weeks to months, depending on the fracture location, severity, and treatment (1). During fracture healing, patients are frequently unable to work for an extended period, which results in substantial socioeconomic costs, especially when healing is delayed (2, 3). Throughout the healing process, patients are usually less mobile and active than they are in their normal lives, be it due to bed rest or partial weight-bearing instructions, pain, or, in more severe cases, the inability to mobilize (4). Numerous negative health effects are associated with reduced physical activity and immobilization, including decreases in muscle and bone mass (4, 5), cardiovascular pathology (6), and deep vein thrombosis (7). It would therefore be desirable if fracture healing could be accelerated and the healing time could be shortened. However, interventions designed to accelerate fracture healing are not part of the current treatment guidelines for bone fractures (8, 9).

An optimized mechanical environment is known to shorten the healing time compared with the extended healing time in less favorable mechanical situations (10). To achieve this optimization, the fracture is usually reduced and immobilized in plaster or cast. In some cases, implants such as plates, nails, screws, or wires are positioned to keep the reduction and fragments in place and to increase stability by fixation. The mechanical demands for optimal healing are known to change throughout the healing process from little to more movement and forces in the fracture gap (10–12). This fact has led to the concept of dynamization (13) and to the development of implants that become less stiff throughout healing (14, 15). In addition to an improvement in the local mechanical situation at the fracture site, mechanical axial micromovement applied via external fixators was shown in the 1980s and 1990s to shorten the time a fracture needed to heal by more than 20% in patients with tibial fractures (16, 17). This facilitation was beyond the shortest healing time that could be accomplished by creating an ideal mechanical situation. The effects of other interventions, such as parathyroid hormone (PTH) and bisphosphonate administration, on acceleration have also been discussed (18, 19). Systematic reviews and meta-analyses concluded that low−intensity pulsed ultrasound (LIPUS) and pulsed electromagnetic fields (PEMFs) may accelerate the time to clinical union (20). For these and several other possible interventions (including drugs, growth factors, and others) that are thought to accelerate fracture healing beyond ideal mechanical conditions, the evidence for their ability to accelerate fracture healing in clinical use has not been recently reviewed. Indeed, a review of all available interventions that have been clinically studied is completely lacking. Furthermore, many of the possible interventions have only been tested in animal studies, and despite their fracture-accelerating effects, they have never been studied in humans with fractures.

**Figure 1:**
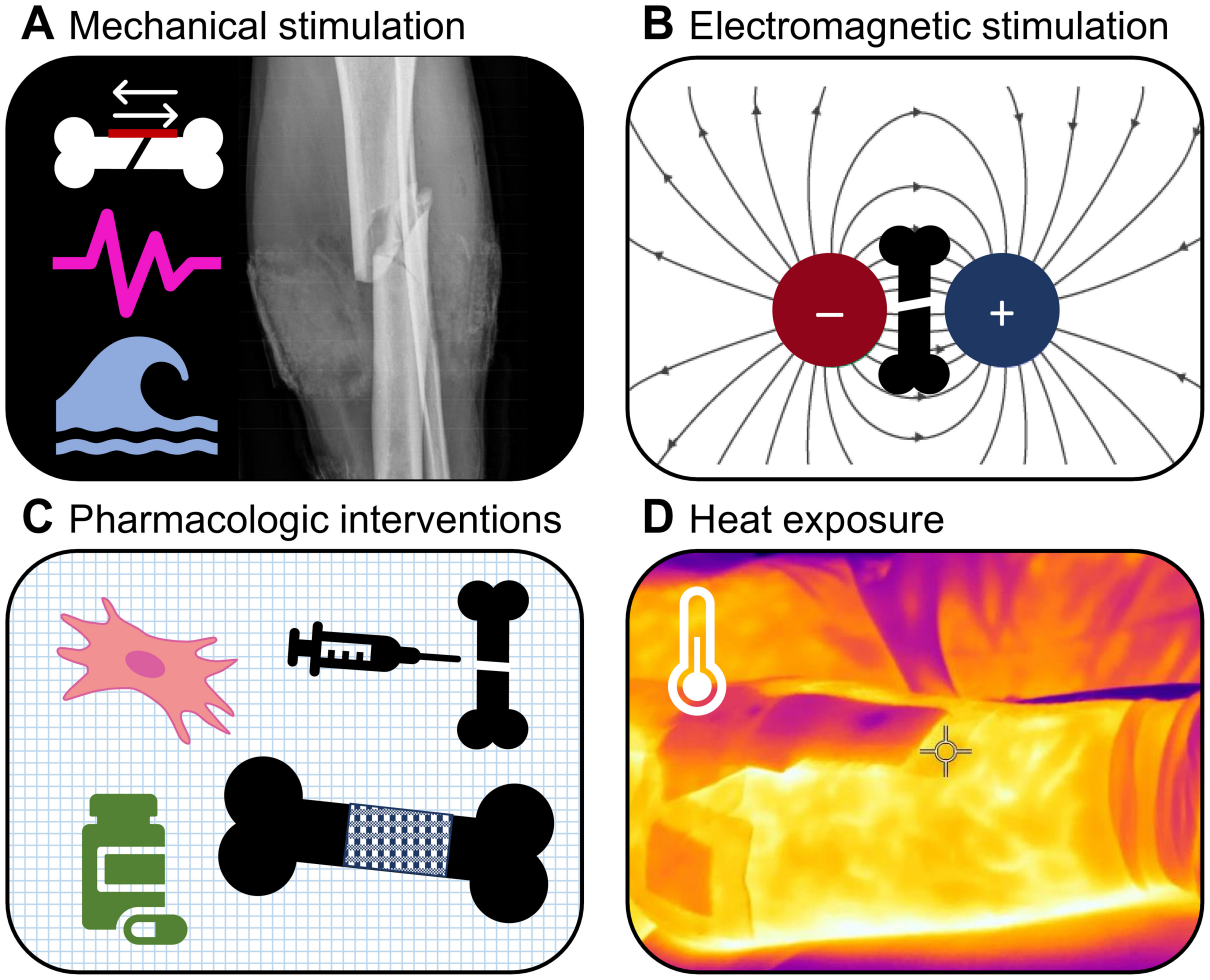
Fields of interventions covered by this review. **(A)** Mechanical stimulation, **(B)** Electromagnetic stimulation, **(C)** Pharmacologic interventions, and **(D)** Heat exposure.

Therefore, the aim of this narrative review article is to provide an overview of the available evidence on methods that shorten the healing time of bone fractures in patients with otherwise healthy bones beyond the effects of an ideal biomechanical, nutritional, and metabolic environment. ‘Patients with otherwise healthy bones’, in this case, are individuals without metabolic or structural bone diseases (such as diabetes or osteoporosis) that slow bone healing and require different and disease-specific therapeutic strategies than those for otherwise healthy patients. This review focuses on clinical evidence from studies involving fracture patients and reports some findings from animal and cell experiments. In addition to mechanical and electromagnetic stimulation, pharmacotherapy, lasers, and other types of stimuli and interventions are reviewed, as well as studies that combine several methods. This review also provides information on knowledge gaps in the field of fracture-healing acceleration, leading to advice on which research needs to be conducted to create an evidence base for clinical guidelines. **Figure 1** illustrates the key fields of interventions covered by this review.

## 2 Mechanical stimulation

### 2.1 Axial micromovement

To improve healing, axial massage of the fracture gap by axial micromovement seems to be a beneficial intervention. The optimal amount of axial interfragmentary movement (IFM) has been reported to be in the range of 0.4–0.5 mm, depending on the distance of the fracture gap (21, 22). In human patients with tibial fractures, 0.5 mm of controlled cyclic axial micromovement in the fracture gap applied via external fixators (23) reduced the healing time by 7.1 weeks (16), which in this study was equivalent to a 23% reduction compared with regular healing (23.7 weeks of healing time with stimulation and 30.8 weeks without stimulation, in groups of 50 and 32 patients). Another study in human patients showed a 6-week reduction in healing time (17), which corresponds to a 21% reduction (23 weeks of healing time with stimulation and 29 weeks without stimulation, in groups of 23 and 22 patients, respectively). The same effect had previously been shown in sheep with osteotomies (24). Similar stimuli may not only be delivered by external fixators but also, in the future, ideally be applied by active implants that provide cyclic shortening and lengthening (15). External fixators, such as those used in the named micromovement studies, are frames that stabilize the fracture outside the skin and are attached to screws and/or wires to connect them to the bone. Among their disadvantages are low patient comfort due to the external frame, risks of pin site infections, and implant breakage or loosening. It is therefore of interest to develop active implants that can deliver axial compression stimuli while being entirely covered by skin (25, 26). In their review on whether biomechanical optimization can shorten fracture-healing time, Barcik et al. (10) concluded that, ideally, stimulation should predominantly be limited to the proliferative phase of healing, and that adequate rest periods are required between applications of stimulation. Thus, it is necessary to adapt the stimulation properties to the individual situation. This may be accomplished by combining the sensor and actor capabilities of the implant in a control circuit (15, 27). As the resultant movement and interfragmentary strain in the fracture gap certainly depend on the fracture geometry and fracture gap width, CT-based finite element simulations may help to determine the stroke of the micromovement needed for the individual fracture (28, 29). Generally, increasing fracture gap sizes are associated with a longer healing process (30). Smaller fracture gaps require stiffer fixation and a lower IFM (31). As strain is applied over a larger distance of fracture fragments, comminuted fractures tolerate relatively greater motion, which reduces the local strain and leads to more timely healing (23).

### 2.2 Vibration

Exposure to whole-body vibration (WBV) by having a patient stand on a vibration platform could in theory deliver a similar stimulus to the fracture gap as the axial micromovement induced by external fixators (16, 17). However, a difference is that force transmission through tissues and joints and muscle contraction may affect the IFM (32). The vibration settings that can be modified include peak-to-peak displacement (amplitude), frequency and acceleration (33). Low-magnitude high-frequency vibration (LMHFV) is defined as vibration with an amplitude from 0.02 mm to 1 mm at 20–90 Hz with less than 1.0 g (gravitational acceleration) (34). In a systematic review on the effect of WBV on fracture healing, Wang et al. (34) reported that stimulation frequencies of 35 Hz and 50 Hz yielded the best results. Their review, however, did not analyze healing acceleration. Wehrle et al. (35) suggested caution when treating fracture patients with LMHFV, as small changes in the settings could considerably change the outcomes. In their study involving mice, a frequency of 35 Hz did not affect fracture healing, whereas a frequency of 45 Hz significantly reduced bone formation and flexural callus rigidity. Wolf et al. (36) tested the effect of exposure to a vibration platform in sheep with a frequency of 50 Hz and an IFM of approximately 0.02 mm magnitude, but did not find an effect on bone healing with these settings. While vibration exposure was shown to normalize fracture healing in diabetic (37) and ovariectomized rats with osteoporosis (38), it did not affect bone healing in healthy rats (37). The vibration settings of these two studies were a peak-to-peak vertical displacement of 1 mm at frequencies of 50 Hz and 60 Hz. The effect of vibration exposure on the speed of fracture healing has not yet been investigated in a study of human patients. However, a clinically relevant effect seems unlikely based on the reported findings.

### 2.3 Ultrasound

LIPUS is defined as acoustic waves with a carrier frequency of approximately 1.5 MHz with pulse clusters of 200 µsec at an intensity of approximately 30 mW/cm^2^ and a repetition rate of the clusters at 1 kHz (39, 40). The devices that usually work by piezoelectric crystals are noninvasively positioned on the skin over the fracture to apply the stimulus for approximately 20 to 30 minutes daily (41). A systematic review concluded that LIPUS did not reduce the time to return to work or the days to weight bearing in fracture patients (42). A recent Cochrane review, due to the statistical heterogeneity of studies, was unable to pool the data for time to fracture union, which means that the authors were unable to analyze the available studies and draw a conclusion (43). The same review concluded that studies did not show an effect on delayed union or nonunion (43). A 2002 review of the effects of LIPUS on time to fracture healing revealed evidence in randomized trials that LIPUS treatment can significantly reduce the time to fracture healing in nonsurgically treated fractures, while there appeared to be no additional benefit after intramedullary nailing with prior reaming (44). In 2014, another systematic review and meta−analysis of randomized controlled trials came to a similar conclusion, reporting that the effect of LIPUS on bone growth stimulation could only be found in patients with nonoperatively treated fractures or fractures of the upper limb (20). The same study revealed that the use of LIPUS for the treatment of diaphyseal fractures accelerated the time to clinical union. More recent clinical trials that tested LIPUS for effects related to fracture-healing acceleration included a study by Murakami et al. (45) who did not observe acceleration after LIPUS treatment of 101 proximal stress fractures of the fifth metatarsal.

Based on the studies and reviews that have been published thus far, it can be concluded that LIPUS at least does not have a decelerating effect on fracture healing, whereas it also does not have a massively accelerating effect that unambiguously appears in most studies. The effect could be greatest in nonoperatively treated fractures and diaphyseal fractures. It also seems that in addition to the common LIPUS settings, further frequencies and settings (intensity, clustering, daily stimulation time) have not been systematically compared in patients regarding their effects on fracture-healing time, and it thus remains unclear if the named LIPUS settings are the optimum or if other settings would deliver more favorable results. Apart from LIPUS, High-intensity focused ultrasound (HIFU) is used to treat bone metastases (46, 47), but to date no clinical study has tested the effect of HIFU in patients with fractures with the aim of accelerating healing (43).

### 2.4 Shock wave therapy

Extracorporeal shock wave therapy (ESWT) applied though single pressure waves of approximately 300 bar activates mechanotransduction (48) and thereby (re-) activates fracture healing via the release of growth factors, such as BMPs, TGF-β, and VEGF (49). Shock waves are generated by electrohydraulic, piezoelectric, or electromagnetic mechanisms (50). Its application led to increases in bone microcirculation in the scaphoid (51). ESWT was shown to accelerate endochondral ossification and fracture healing in a rat femur delayed-union model (52). It also seems to be effective in stimulating the healing process in delayed unions and nonunions, where it has convincing evidence of a beneficial effect (53). However, studies to generate evidence for an accelerating effect on human fracture healing are lacking. It would be beneficial, if large randomized clinical interventional studies could assess the effect of ESWT on fracture-healing speed in human fracture patients.

## 3 Electromagnetic stimulation

**Figure 2:**
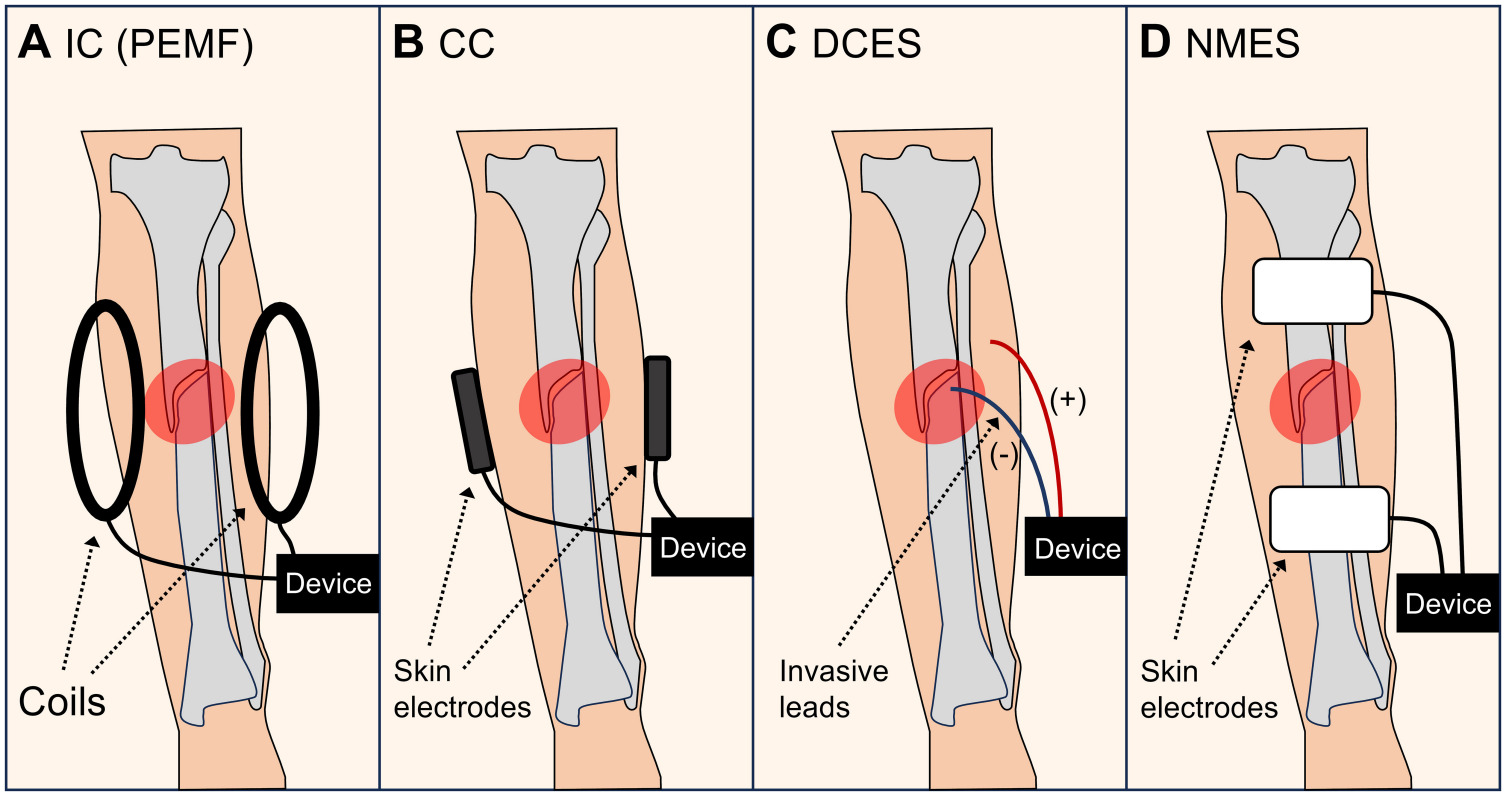
Types of electromagnetic stimulation. **(A)** Inductive coupling (IC), such as pulsed electromagnetic fields (PEMF). **(B)** Capacitive coupling (CC). **(C)** Direct current electrical stimulation (DCES). **(D)** Neuromuscular electrical stimulation (NMES).

Bone is a bioelectric tissue in which charges, streaming potentials, and piezoelectricity can be observed (54, 55). Several modes of electromagnetic stimulation seem to have an effect on fracture-healing speed (56). The options differ widely in terms of their properties and intensities and include exposure to electromagnetic fields (i.e., capacitive coupling (CC) or inductive coupling (IC)), direct electrical stimulation with a current running through the fracture (direct current electrical stimulation (DCES)), or indirect fracture stimulation by neuromuscular electrical stimulation (NMES). **Figure 2** illustrates the different types of electromagnetic stimulation. To promote regeneration, electromagnetic fields can be applied in a pulsed, constant, or alternating manner (57). In a recent review on the effects of electrical stimulation on acute fractures (not specifically on fracture acceleration), Nicksic et al. (58) reported a lack of evidence and study comparability and suggested a protocol to conduct repeatable, well-reported studies. This is particularly needed due to the large variety of possible settings. In their review addressing the question of why more surgeons do not use electromagnetic stimulation despite its effectiveness, Bhavsar et al. (59) pointed out that surgeons would likely be open to using this treatment if advancements in the technology are able to provide an easy-to-use, cost-effective method to deliver electrical stimulation to their fracture patients. The authors also noted that complications included skin irritation and infections, pain, dislocation of the device, device failure, and poor patient compliance.

### 3.1 Noninvasive application of electromagnetic fields

PEMF is a type of IC that is applied via coils outside the body. It is thought to induce osteogenic differentiation of osteoprogenitor cells, and to promote angiogenesis, and bone mineralization (60). It has also been shown to induce vasodilation (61). Combined electric and magnetic field therapy in a sheep model showed enhanced bone healing, resulting in better new bone structure, callus morphology, and superior biomechanical properties compared to those of a nonintervention control group (62). Among the options for electromagnetic field application in human fracture patients, the most clinical evidence seems to exist for PEMF. Several studies have been conducted in human fracture patients. The time to cast removal was significantly shorter in patients with distal radial fractures treated with PEMF (33 ± 5.9 days) than in patients in the sham group (39.8 ± 7.4 days), equivalent to a 17% reduction in time (63). In 40 patients with closed or Grade-1 open tibial fractures, Fontanesi et al. (64) reported a significant reduction in time to union from an average of 109 to 86 days (a difference of 25 days, a reduction of 21%). In these successful studies, the hardware and settings were as follows: the magnetic field was 2–2.8 mTesla, and each impulse lasted 1.3 milliseconds. The tension induced in the calibrated probe was 2.5 ± 1 mVolt. In acute distal radial fractures, Factor et al. (63) reported a 17% reduction in the fracture healing time. For this study, a PEMF device called a fracture healing patch (FHP) (Pulsar Medtech Ltd., Bnei Brak, Israel) was used with a pulse frequency of 20 kHz, a cycle frequency of 10 Hz, and a pulse intensity at the fracture site of between 0.05 and 0.5 mTesla. In addition, Wahlström (65) studied the effect of electromagnetic fields with extremely low frequency (EMF of ELF) with an alternating magnetic field with a frequency of 1–1000 Hz and a magnitude of 4 Gs. Progress in the bone healing of 50 women with distal radius fractures was observed with scintigrams (accumulation of 99mTechnetium). The findings indicated a significantly shorter time until normal scintigram values were achieved in the stimulated group. According to a systematic review and meta−analysis of randomized controlled trials, PEMF significantly shortened the time to radiological union for acute fractures treated without surgery and for acute fractures of the upper limb (20). Thus, exposure to electromagnetic fields seems to be a useful option for accelerating fracture healing.

CC usually involves the placement of electrodes on the skin on opposite sides of the fracture, and the application of alternating currents. In human patients with vertebral fractures, CCs showed faster resolution of vertebral bone marrow oedema, less pain and reduced pain-medication consumption (66, 67). Clinical studies on the effect of CC on long-bone nonunions have been published, but to date (68, 69), to the author’s knowledge, no study on CC has focused on fracture-healing speed or acceleration in long-bone fractures. One reason may be that due to the extensive soft tissue mantle in humans, external electromagnetic field application has the disadvantage of field intensities that are mostly too low to achieve the desired effect in the bone, which is why invasive and implant-based DCES options should be further explored (70).

### 3.2 Treatment with direct electric currents

Apart from noninvasive electromagnetic field application, electromagnetic fields can also be applied by implanted electrodes (58, 70, 71). This application mode allows the delivery of the electromagnetic field in a more optimized intensity range for the local tissue. Finite element simulations of the electromagnetic field distribution are also beneficial for determining the required settings before a clinical trial, as these settings highly depend on the local tissue properties (72). In addition, the success of electromagnetic stimulation was shown to depend on the local oxygen tension, which means that it only works with adequate blood supply (73).

DCES treatment is usually applied at dosages between 5 and 40 μA (59). In 24 human patients with tibial fractures treated with a Hoffmann external fixator, Jorgensen (74) used insulated bone screws as electrodes to induce a pulsating voltage at 1 Hz and approximately 0.7 V and 40 µA. A voltage of 1.5 volts or more was considered painful for the patients. Changes in the stiffness of the fracture were recorded through the Hoffmann apparatus. The results showed a 30% decrease in fracture-healing time in the intervention group (2.4 months) compared to the control group (3.6 months). With a fully implanted device for direct current fracture stimulation, named ‘direct current bone growth stimulation’ (DCBGS, constant current at 20 µA, 3 V), Paterson et al. (75) confirmed the short period until healing (average of 16 weeks) in 84 patients with delayed union or nonunion when using the device. Many further DC clinical studies were conducted with the aim of treating nonunion (59), but the effects of healing acceleration were not followed up for translation into clinical practice. As the early findings appear to be extremely promising, large clinical studies should now test the effects of the application of modern DC devices on fracture-healing time. As the invasiveness of this intervention is certainly a disadvantage, it may be an option to add this functionality to smart implants such as plates or nails, when they are implanted anyway (15). For example, an implantable electric pulse stimulator that combines a hybrid tribo/piezoelectric nanogenerator with a conductive bioactive hydrogel was recently presented and very promising data from experiments with mice were obtained (76).

### 3.3 Neuromuscular electrical stimulation

NMES can be used to induce involuntary muscle contractions with a mechanical effect on fracture biomechanics, including strain and movement in the fracture gap (77, 78). These effects, however, are difficult to standardize, as the individual muscle contractile properties, which are known to change with immobilization, need to be taken into account (79, 80). NMES has been shown to enhance fracture healing in mice (81), but studies on the effects of NMES use on fracture-healing speed in humans are lacking. It would be of interest to explore this possible intervention, as it is noninvasive and might be helpful, especially when combined with other methods. NMES may be particularly beneficial in bed-ridden patients, e.g., those with spinal cord injury, as they lack movement and strain in the fracture.

## 4 Pharmacologic interventions

Pharmacologic interventions can either be applied systemically or directly to the fracture site (injection or intraoperative application) (82, 83). Local drug delivery by injecting a medication into the fracture gap could be an option for conservative treatment or for application when delayed healing occurs following surgery. In a review, Per Aspenberg listed and discussed drugs that impair or improve fracture healing (84). The drugs he listed that improve fracture healing were growth factors (fibroblast growth factors (FGF) 1 and 2, transforming growth factor 1 (TGF-1), growth hormone (GH), and bone morphogenetic proteins (BMPs)), PTH, selective prostaglandin agonists, statins, and beta blockers. In another review on fracture repair acceleration from 2013 (85), he named bisphosphonates, BMP and PTH as potentially accelerating fracture healing, but a clear conclusion at that time was not possible due to the need for more and better studies. Nielsen & Low (86) published a review focusing on bone-targeted ligands to accelerate fracture healing and highlighted dose-limiting toxicities such as hypercalcaemia with the systemic use of many osteoporotic drugs when administered to accelerate fracture healing, which can be reduced with selective drug delivery.

### 4.1 Growth factors

Accelerated fracture healing was observed after traumatic brain injury (TBI) in patients whose serum concentrations of BMP-2, FGF-2, IL-1β, and platelet-derived growth factor (PDGF) were increased (87). Furthermore, GH, interleukin-6 (IL-6), and prolactin levels were elevated in TBI patients who had increased callus formation (88). In TBI patients, the time to callus formation was also shortened (89). These acceleration effects appear clinically very impressive and relevant. The injection of the same factors in patients without TBI may lead to the same effects, which should be tested in clinical intervention studies. In fracture patients, brief periods of ischaemia generated by pneumatic cuff compression were also shown to accelerate bone fracture healing, likely through the release of BMP-2 and other growth factors (90). This promising method requires further well-designed clinical studies.

The injection of mesenchymal stem cells that locally produce growth factors for the acceleration of fracture healing has been discussed and studied. In mice, injections of mesenchymal stem cells (MSCs) that overexpress basic fibroblast growth factor (bFGF) accelerated fracture healing (91). Encapsulated nerve growth factor (β-NGF) in injectable microrods in mice led to a significant increase in the percentage of bone in the fracture callus, trabecular connective density, and bone mineral density relative to those in controls (92). In addition, stimulation of the Wingless and Int-1 (Wnt) pathways by Wnt1 was found to significantly accelerate fracture healing by enhancing bone formation in mice (93). In early experimental studies, inhibition of Ca2+/calmodulin (CaM)-dependent protein kinase kinase 2 (CaMKK2) was shown to accelerate endochondral ossification, resulting in more rapid and efficient fracture healing (94). However, no studies with patients have assessed the effect of these growth factors on fracture-healing speed. The available clinical studies on the amelioration of fracture-healing by MSCs in patients (not specifically focusing on healing acceleration) were summarized in a systematic review and meta-analysis by Yi et al. (95), which revealed no decrease in healing time in the MSC group. The included studies with human patients differed largely in their patient collectives and modes of MSC application (96–99). A fracture-accelerating effect thus seems possible, however, more sufficiently large and prospective intervention studies in human patients are required to be able to reach a conclusion. Bone morphogenetic proteins (BMPs) are thought to initiate fracture healing and to thereby reduce nonunions (85). Local intraoperative application of mineral-coated microparticles loaded with VEGF and BMP-2 induced the healing of atrophic nonunion in mice (100). In rats, a single percutaneous injection of recombinant human BMP-2 accelerated fracture repair (101). Among the larger studies in patients is a clinical trial with 277 patients with open tibial fractures treated with reamed intramedullary nail fixation that was not significantly accelerated by the addition of rhBMP-2 delivered by an absorbable collagen sponge (102). A Cochrane review on the effects of intraoperative application of BMP on fracture healing concluded that there is limited evidence that BMP is more effective than control treatment, and the efficacy of BMP for the treatment of nonunion remains unclear (103). The injection of BMPs may be associated with ectopic bone formation, which has been identified as a clinical complication (104). It can be concluded, with uncertain evidence, that BMPs at least do not markedly accelerate fracture healing but rather have a lesser effect, if any.

Further clinical studies in patients are needed to quantify the effects of the above growth factors and combinations of several of growth factors, also considering the mode of application on healing time.

### 4.2 Medications

In osteoporosis, bone healing is generally slower, and bisphosphonates have been shown to facilitate healing in terms of normalizing the fracture-healing speed (18, 85, 105). However, there is no indication that bisphosphonates also accelerate healing in patients without osteoporosis (85). PTH, marketed as the osteoporosis drug teriparatide, increases bone remodeling by increasing the number and activity of osteoblasts, thereby affecting early callus formation (85). PTH had no effect on the chondroid phase of fracture healing, but an effect was observed once bridging bone developed (106). Although the evidence for fracture-healing acceleration is limited, there seems to be at least a promising beneficial effect that makes PTH an interesting candidate (18, 19, 85, 107, 108). In 65 postmenopausal women with pelvic fractures, PTH accelerated fracture healing by more than 30% (7.8 weeks in the PTH group compared with 12.6 weeks in the control group) and improved functional outcomes (19). In 102 postmenopausal women with distal radial fractures, daily placebo, 20 µg teriparatide or 40 µg teriparatide (34 patients in each group) resulted in a shorter healing time with teriparatide 20 µg but not with 40 µg teriparatide, compared with placebo (18). Intermittent PTH accelerated stress fracture healing more effectively following cessation of bisphosphonate treatment (109).

Acidic oligopeptides such as desatinib have been proposed for the acceleration of fracture healing, as their systemic side effects could be very low when targeted to the fracture site (selective drug delivery), while a strong effect on healing time is expected with local administration; however, clinical studies involving fracture patients are currently unavailable (86, 110). Other drugs that have been considered to accelerate fracture healing include selective prostaglandin agonists (111), cyasterone (112), statins, and anti-sclerostin antibodies (113), but there is a lack of evidence from clinical studies in patients. In animal studies, amlodipine (114), cilostazol (115), L-arginin (116), sildenafil (117), and metformin (118) were shown to facilitate fracture healing. Aditionally, irisin (119), tumeric acid (120), aqueous extract of ***Prunus dulcis*** (121), and the Chinese acupuncture and herbal formula powder prescription ‘Zhèng Gŭ Zĭ Jīn Dān’ (122) are thought to accelerate fracture healing, but this has not yet been shown in clinical studies with patients and with an appropriate study design.

In summary, PTH is currently the medication with the best evidence for accelerating fracture healing. When considering pharmacotherapy to accelerate fracture healing, side effects must be considered. These can be significant compared to the side effects of mechanical or electromagnetic interventions; therefore, patient-specific comorbidities and current medications need to be considered much more for pharmacological interventions than for other interventions.

## 5 Heat exposure

Light amplification by stimulated emission of radiation (laser) is a method developed in the 1960s that generates and emits coherent and focused light. It has various applications in medicine, including for analgesia (123, 124). Laser light with a power of more than 500 mW is called a high-intensity laser (HIL), and it acts by producing heat in the tissue. Lasers with a power of less than 500 mW are called low-level lasers (LLLs). The latter are usually applied at wavelengths of 600 to 1000 nm and do not produce heat but still seem to have effects on pain, inflammation, tissue repair and regeneration by stimulating cellular and enzymatic processes, including those involving stem cells (125). These effects seem to occur in the early but not in the later stages of fracture healing (125). In rats with bone defects, low-level laser therapy (LLLT) accelerated the development of newly formed bone during the initial phase of bone healing (126). A combination of bone marrow aspirate (BMA) and LLLT was reported to enhance bone healing in rats (127). Only a few studies have investigated the effects of HIL therapy on fracture healing. Kim et al. (128) showed that high-intensity Nd: YAG laser irradiation significantly increased new bone formation in bone defects by approximately 45%. However, in human patients, such studies are rare. Among the few clinical studies that have tested LLLT on closed bone fractures of the wrist and hand, LLLT improved the VAS score, Quick-DASH score, and hand and finger grip strength (129). However, no clinical studies in patients have assessed the effects of LLLT or HIL on fracture-healing speed. Based on the promising findings from animal studies, such trials appear highly desirable. Aditionally, clinical studies with other heat sources, such as transdermal heat application, are needed.

## 6 Combination of interventions

**Figure 3:**
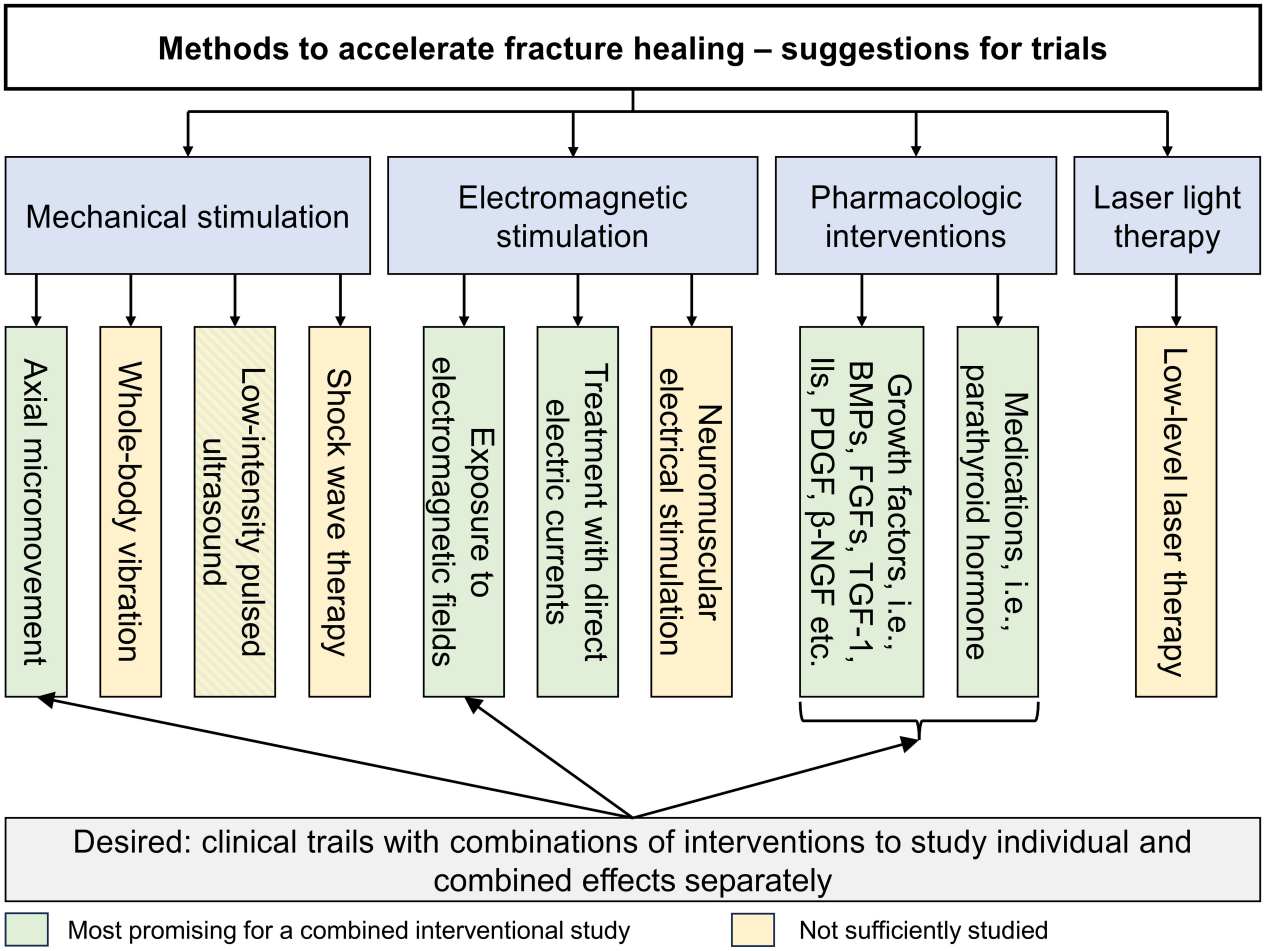
Overview of methods and suggestion for clinical trials.

**Figure 3** provides an overview of the available methods and a possible combination of methods as a priority suggestion for clinical trials. In theory, a combination of interventions that have been shown to accelerate fracture healing could decrease the healing time even more than one intervention alone, especially if their mechanisms of action differ. Several studies have combined interventions to examine their joint effects on fracture-healing speed, such as a combination of stem cells and PTH (130) or a combination of human PTH and LIPUS (131). However, none of these studies were designed to assess the individual and combined effects separately or to compare them to a control group. This is the desirable study design for determining whether there is an advantage in administering two or more interventions at once, compared to only one intervention. Based on the findings collected in this review, combinations of axial micromovement and electromagnetic stimulation and the therapeutic application of growth factors and/or PTH could be interesting to explore in such studies. In addition, the ideal settings (i.e., frequencies, intensities) and exposure times need to be explored for each method alone and for several combined. These may differ depending on which and how many interventions are combined methods. Such studies are difficult to conduct, as they need large patient groups and suitable outcome measures. They are, however, urgently needed in the clinic.

## 7 Discussion

**Figure 4:**
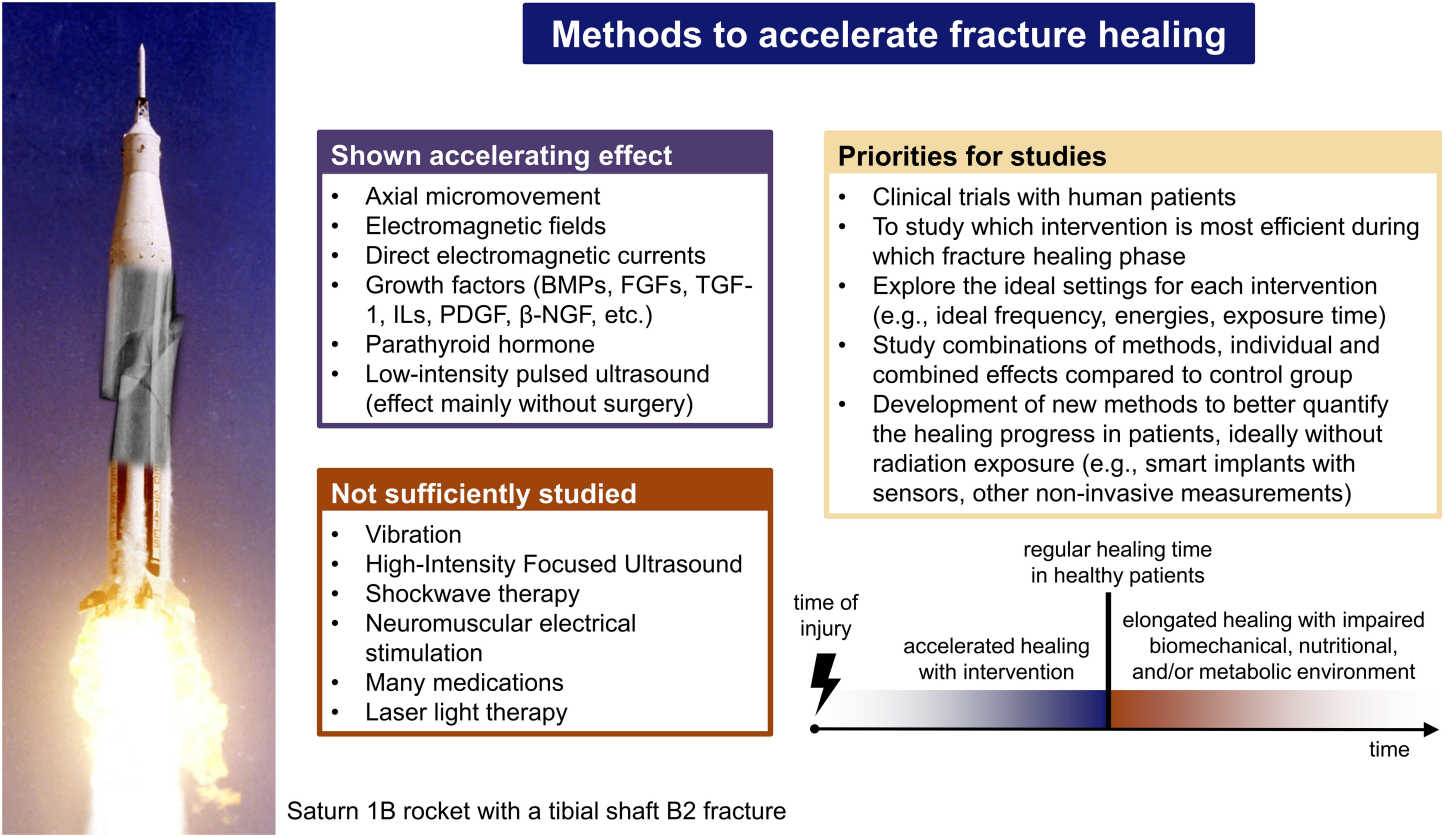
Summary of the findings of this review.

The main findings of the present review are summarized in **Figure 4**.

Fracture healing occurs in several phases over several weeks (23, 132). These include the acute inflammatory response, recruitment of mesenchymal stem cells, generation of a cartilaginous and periosteal bony callus, revascularization and neoangiogenesis at the fracture site, and mineralization and resorption of the cartilaginous callus, followed by bone remodelling (1). In regular healing, numerous cellular components are involved, including growth fractures and cytokines that induce vascularization of the fracture callus within 2 to 5 weeks (133). New blood vessels provide cells, hormones, and nutrients for callus development from avascular cartilaginous tissue to mineralized woven bone (134). Direct or indirect fracture healing may occur (1), also called contact healing when the bone ends are in direct contact and gap healing when they are not directly apposed. On average, the healing time (also known as the union time) is increased in smokers (135), in patients with osteoporosis (136), in patients receiving bisphosphonate therapy (137), in patients in a hypoxic environment, such as at high altitude (138), in patients with more complex fractures, including those associated with compartment syndrome (139), and in older patients and patients with several comorbidities (140).

In the clinical routine, in addition to clinical appearance, X-ray radiography and computed tomography (CT) scans are used to objectively monitor the progress of fracture healing (141). These two X-ray based imaging techniques are, however, associated with health risks connected to radiation exposure (142). Nonetheless, in some cases, ultrasound imaging can be used with limited informative power (143, 144). In addition to radiation exposure, X-ray-based imaging has the disadvantage of showing healing progress with a time lag, as it reflects only the progress in calcification and not the actual improvements in stiffness (145). The development of additional methods to measure the progress of fracture healing that can be used in the clinical environment is highly desirable (146). These interventions are also urgently needed, as they have not become part of the standard of care to date, despite the highly promising evidence presented in the present review. Moreover, no inexpensive and easy-to-use alternative to radiography-based methods for monitoring fracture healing has made its way into clinical routine. However, gait and motion analyses are currently being explored for this purpose (147), and the development of implants with sensors that measure changes in stiffness has recently shown some innovations (15, 25, 148). In addition, bioimpedance and perfusion measurements seem to be promising (133, 149, 150). Among the current ideas under development are also autonomous implants with combined sensing and acting capabilities that have a feedback loop and that apply interventions depending on live measurements to accelerate healing (15). Pulsatile electrical stimulation on cells was combined with such a real-time readout by impedance sensing to design a control loop as an example of how an active implant could be regulated in the future (27). Patient compliance is a problem when the intervention requires active patient cooperation (151). The automated application of an intervention by an implant or the automated release of a medication could improve this aspect. Shorter fracture-healing times could reduce the likelihood of secondary fracture displacement and complication rates, leading to changes in clinical practice. In some cases, these include the decision of when to operate and when to choose conservative treatment. While fractures with displacement in the joint line almost always require surgery to avoid osteoarthritis, simple closed shaft fractures, which are now often operated on to reduce immobilization-related complications, could be treated conservatively in more cases if it turns out that some of the above interventions allow fracture-healing times to be reduced. This could be particularly relevant for patients who need to return to work as quickly as possible, as well as for geriatric patients.

Fracture healing in the clinical setting has many influencing factors for the treating clinical team, which challenges the translation from simplified preclinical findings into clinical practice. Among these challenges are the individuality of patients and their compliance (151), economic pressures, and structural issues that often make it difficult to conduct large randomized, longitudinal clinical trials in hospitals. It is therefore of the highest priority to support and prioritize this kind of research and to conduct the clinical studies suggested in the present review in human patients. The acceleration of fracture healing shows a large gap between the very clear preclinical evidence and the clinical studies needed to translate these findings into clinical practice, and generate a real benefit for the patients.

## 8 Conclusions

In summary, despite the massive health and socioeconomic benefits of shorter fracture-healing times, there is a large research gap regarding the actual benefits of possible interventions to reduce these healing times in patients. The most promising methods to accelerate fracture healing appear to be the application of axial micromovement, electromagnetic stimulation with electromagnetic fields and direct electric currents, and the administration of growth factors, and PTH. The optimal stimulation settings with the most ideal results, e.g., frequencies and energies, have not yet been sufficiently researched. As a combination of these interventions could decrease the healing time further than one intervention alone, especially if their mechanisms of action differ, clinical multicenter studies involving human patients are needed to assess the individual and combined effects compared with a control group. To make it easier to conduct such studies, the development of new methods that allow better quantification of the progress of fracture healing is needed.

## Author contributions

BG: Conceptualization, Funding acquisition, Project administration, Visualization, Writing – original draft, Writing – review & editing.
